# The effects of aging on the functional and structural properties of the rat basilar artery

**DOI:** 10.14814/phy2.12031

**Published:** 2014-06-11

**Authors:** Nihal Tümer, Hale Z. Toklu, Judy M. Muller‐Delp, Şehkar Oktay, Payal Ghosh, Kevin Strang, Michael D. Delp, Philip J. Scarpace

**Affiliations:** 1 Geriatric Research, Education, and Clinical Center, North Florida/South Georgia Veterans Health System, Gainesville, Florida, USA; 2 Department of Pharmacology and Therapeutics, University of Florida, Gainesville, Florida, USA; 3 Department of Physiology and Functional Genomics, University of Florida, Gainesville, Florida, USA; 4 School of Dentistry, Marmara University, Istanbul, Turkey; 5 Department of Applied Physiology and Kinesiology, University of Florida, Gainesville, Florida, USA; 6 Department of Psychiatry, University of Florida, Gainesville, Florida, USA

**Keywords:** Acetylcholine, aging, antioxidant capacity, basilar artery, endothelin, NO, papaverin, vascular smooth muscle, vasoreactivity, vertebrobasilar insufficiency

## Abstract

Aging leads to progressive pathophysiological changes in blood vessels of the brain and periphery. The aim of this study was to evaluate the effects of aging on cerebral vascular function and structure. Basilar arteries were isolated from male Fischer 344 cross Brown Norway (F344xBN) rats at 3, 8, and 24 months of age. The basilar arteries were cannulated in the pressurized system (90 cm H_2_O). Contractile responses to KCl (30–120 mmol/L) and endothelin‐1 (10^−11^–10^−7^ mol/L) were evaluated. Responses to acetylcholine (ACh) (10^−10^–10^−4^ mol/L), diethylamine (DEA)‐NONO‐ate (10^−10^–10^−4^ mol/L), and papaverin (10^−10^–10^−4^ mol/L) were assessed to determine both endothelium‐dependent and endothelium‐independent responsiveness. Advanced aging (24 months) decreased responses of the basilar artery to both the contractile and relaxing agents; whereas, DEA‐induced dilation was significantly higher in the 8‐month‐old group compared with the younger and older groups. The arterial wall‐to‐lumen ratio was significantly increased in 24‐month‐old rats. Smooth muscle cell count was also decreased in old rats. These findings indicate that aging produces dysfunction of both the endothelium and the vascular smooth muscle in the basilar artery. Aging also alters wall structure of the basilar artery, possibly through decreases in smooth muscle cell number and concomitant hypertrophy.

## Abstract (precis)

The purpose of this study was to determine the effects of advancing age on the structure and vasomotor responses of the basilar artery as well as the serum antioxidant capacity. Advanced aging (24 months) decreased responses of the basilar artery to both the contractile and relaxing agents, whereas, DEA‐induced dilation was significantly higher in the 8‐month‐old group compared with the younger and older rats. The arterial wall‐to‐lumen ratio was significantly increased in 24‐month‐old rats. Smooth muscle cell count was also decreased in old rats. Our findings demonstrate that aging is associated with functional impairment in endothelium‐dependent and ‐independent relaxation responses and contractility in the basilar arteries, and these diminished responses are accompanied by structural remodeling and decreased antioxidant capacity of the serum.

## Introduction

Aging is associated with increased risk of cardiovascular disease. The prevalence of hypertension, atherosclerosis, and other cardiovascular complications increase with age. Recent studies have highlighted the structural and functional changes in the vasculature and focused on the mechanisms of vascular aging (Bachschmid et al. 2013). The increase in intima‐media thickness and vascular stiffness; the reduced number of smooth muscle cells and production/ availability of endothelial nitric oxide (NO) and other vasoactive agents; and the increased oxidative stress are proposed mechanisms that impair vascular homeostasis. These subtle changes conspire to trigger vascular dysfunction and lay the foundations for cardiovascular diseases (Muller‐Delp 2011; Golbidi and Laher 2013).

It is well known that the development of atherosclerosis and disturbances in cardiac output compromise the cerebral circulation during aging. To compensate for this lifelong slow reduction of cerebral blood supply, the resistance vessels in the brain gradually diminish their vasomotor tone in order to maintain relatively constant perfusion. Nevertheless, the vasomotor reserve capacity gradually diminishes and the brain is less effectively protected against acute fluctuations in cardiovascular parameters (Kalvach et al. 2007).

The basilar artery is the most important artery in the posterior cerebral circulation. It supplies blood to the medulla, cerebellum, pons, midbrain, thalamus, and occipital cortex. In the United States, approximately one fourth of strokes and transient ischemic attacks occur in the vertebrobasilar region. In the cases of stroke due to acute basilar artery occlusion, the mortality rate is significantly higher when compared to all stroke cases (Gudiene et al. 2011).

Vertebrobasilar insufficiency, or vertebral basilar ischemia (VBI, also called Beauty parlour syndrome), refers to a temporary set of symptoms due to decreased blood flow in the posterior circulation of the brain. The symptoms due to VBI vary according to which portions of the brain experience significantly decreased blood flow (Lekic and Ani 2012). The symptoms may vary from vertigo, headaches, sleep disturbances, pupillary and oculomotor abnormalities, dysarthria, and dysphagia to quadriparesis (Mattle et al. 2011). Likewise, aging has been shown to diminish perfusion of the posterior cerebral circulation in old rats relative to that in juvenile and young adult animals (Ohata et al. 1981; Salter et al. 1998).

The effect of aging on aorta and other peripheral vessels is widely studied in several rat strains; however, little is known regarding basilar artery function with age in the Fischer 344x Brown Norway (F344xBN) rat strain, a commonly used model for aging. Therefore, the purpose of this study was to determine the effects of advancing age on the structure and vasomotor responses of the basilar artery as well as the serum antioxidant capacity.

## Methods

### Animals

Male F344xBN rats 3, 8, 24 months old (*N* = 6/age group) were obtained from Harlan Labs (Indianapolis, IN). Upon arrival, rats were examined and remained in quarantine for 1 week. Animals were cared for in accordance with the principles of the Guide to the Care and Use of Experimental Animals and protocols were approved by the University of Florida Institutional Animal Care and Use Committee. Rats were maintained on a 12:12 h light–dark cycle and provided food (AIN93 diet) and water ad libitum throughout the experimental protocol.

### Serum antioxidant capacity assay

Total antioxidant capacity of serum was measured using the total antioxidant capacity kit (Abcam, Cambridge, UK) according to the manufacturer's instructions. Briefly, plasma was allowed to reduce Cu^2+^ for 1.5 h at room temperature. Reduced Cu^+^ was chelated with a colorimetric probe and absorbance was measured at 570 nm. Results were expressed as trolox equivalent according to a trolox standard curve.

### Microvessel preparation

Rats were anesthetized (isoflurane 3%/O_2_ balance) and euthanized by the removal of the heart. The brain was rinsed and placed in cold (4°C) physiological saline solution (PSS) containing 145.0 mmol/L NaCl, 4.7 mmol/L KCl, 2.0 mM CaCl_2_, 1.17 mmol/L MgSO_4_, 1.2 mmol/L NaH_2_PO_4_, 5.0 mmol/L glucose, 2.0 mmol/L pyruvate, 0.02 mmol/L EDTA, 3.0 mmol/L MOPS buffer, and 1 g/100 mL bovine serum albumin (BSA), pH 7.4. The basilar arteries were isolated with the aid of a dissection microscope (Olympus SZH10, Tokyo, Japan). The arteries were transferred to a Lucite chamber containing PSS equilibrated with room air. The ends of the artery were canulated with micropipettes and secured with nylon sutures. The chamber containing the cannulated artery was then placed on an inverted microscope (Olympus IX71, Tokyo, Japan) equipped with a video camera and micrometer (Panasonic BP310; Texas A&M Cardiovascular Research Institute) to measure intraluminal diameter. The basilar arteries were then pressurized at 90 cmH_2_O (≅ 66 mmHg) with two hydrostatic columns (Faraci and Heistad 1990). Arteries unable to hold pressure due to leaks or branches were discarded. Arteries without leaks were warmed to 37°C and allowed to equilibrate for 40 min before beginning the assessment of vasoconstrictor or vasodilator responses.

### KCL‐induced vasoconstriction

A concentration–response curve to the nonreceptor agonist, KCl (30, 50, 80, 100, 120, 150 mmol/L, isotonic substitution for NaCl) was given in 2‐min intervals.

### Endothelin‐induced vasoconstriction

To determine whether aging alters sensitivity and/or maximal responses to a receptor‐mediated agonist, a concentration–response curve to endothelin‐1 (ET) was generated. Changes in diameter were measured in response to the cumulative addition of ET (1 × 10^−11 ^mol/L–1 × 10^−7 ^mol/L; 2‐min intervals) to the vessel bath.

### Acetylcholine vasodilation

Endothelium‐dependent dilation to ACh was evaluated by the addition of ACh to the bath in incremental doses every 2 min (1 × 10^−10 ^mol/L to 1 × 10^−4 ^mol/L).

### DEA‐NONO‐ate vasodilation

Endothelium‐independent dilation was evaluated by the addition of a nitric oxide donor, diethylamineNONO‐ate (DEA‐NONO‐ate) to the bath in incremental doses every 2 min (1 × 10^−10^M to 1 × 10^−4 ^mol/L).

### Papaverin vasodilation

Responsiveness to papaverin, a direct smooth muscle vasodilator that signals through cyclic AMP, was evaluated by the addition of papaverin to the bath in incremental doses every 2 min (1 × 10^−10 ^mol/L to 1 × 10^−4 ^mol/L).

### Calculations

For contractility studies the following formula was used.



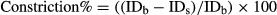



where ID_max_ is the maximal diameter recorded in calcium‐free PSS, ID_s_ is the steady‐state diameter recorded at 90 mmHg, and ID_b_ is the baseline diameter recorded after incubation in inhibitor and immediately prior to the initiation of the concentration–response relationship. Maximum vasodilation (ID_max_) was obtained by the addition of sodium nitroprusside (SNP) (10^−4 ^mol/L), a direct NO donor, to the calcium‐free PSS bath at the end of the experiments.

For vasodilation experiments, a minimum of 15% spontaneous tone was necessary prior to assessment of concentration–response curves.



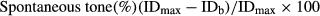



The relaxation response was calculated according to the following formula:



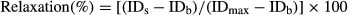



### Hemotoxylin & Eosin staining of basilar artery

Basilar arteries were cannulated, pressurized, and fixed in formalin. Fixed arteries were placed in optimal cutting temperature (OCT) compound and stored at −80°C, until cutting into 5 *μ*m sections. Sections were then stained by Hemotoxylin & Eosin (H&E) method.

Wall thickness, endothelial thickness, media‐intima thickness and number of smooth muscle cells were determined under the microscope (Axiovert 40 CFL, Zeiss, Germany) connected to a computerized system using the Image J/Lab program.

### Statistical analysis

Statistical analysis was carried out using GraphPad Prism 5.0 (GraphPad Software, San Diego, CA). Each group consisted of six animals. All data were expressed as means ± SEM. Sigmoidal dose–response curves drawn and groups/doses were compared with two‐way analysis of variance (ANOVA) followed by Bonferroni post hoc tests. The histological evaluation was done by one‐way ANOVA followed by Tukey's multiple comparison. Values of *P *<**0.05 were regarded as significant.

## Results

### Contractility responses

**Figure 1.**
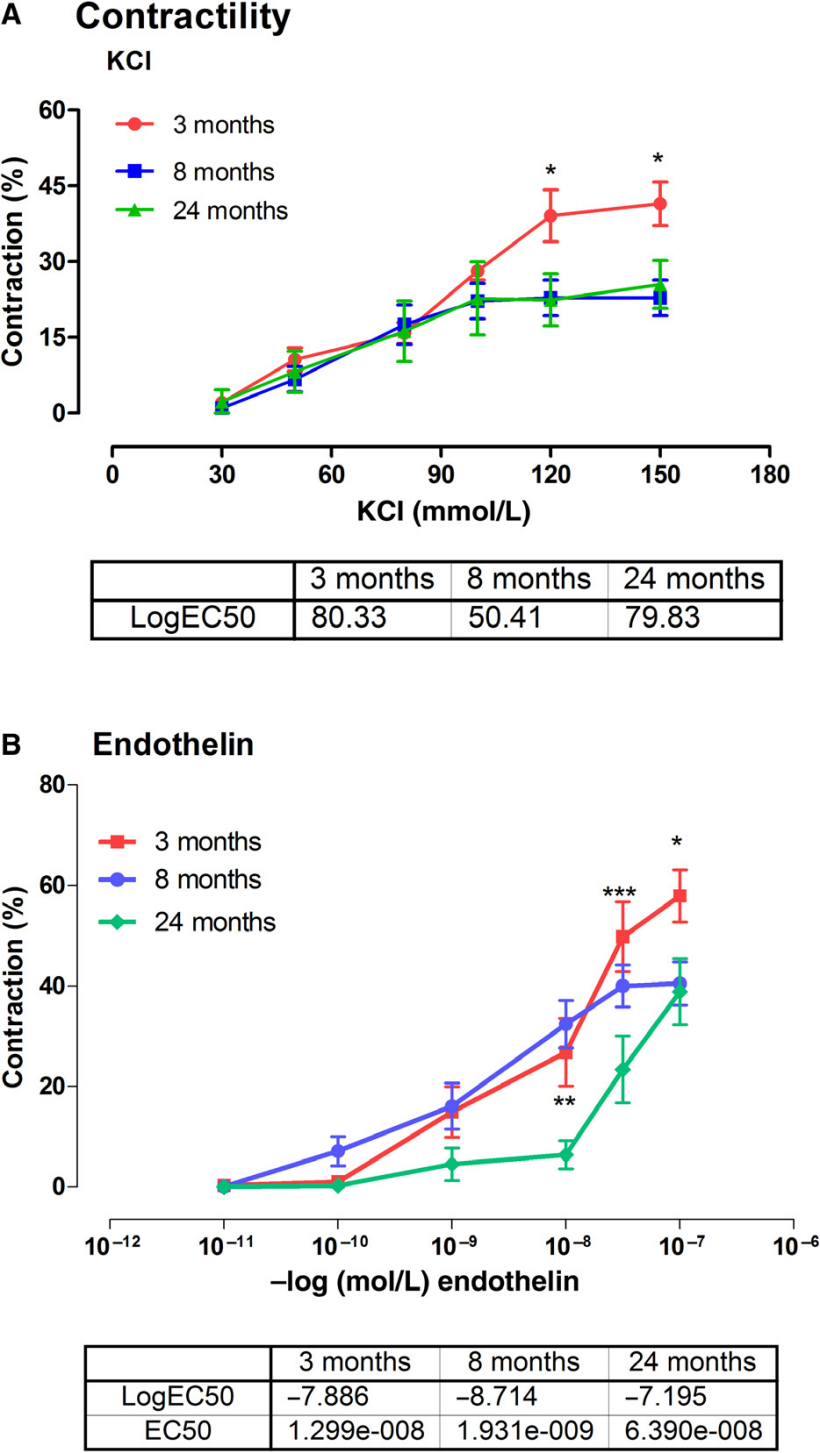
Contractile responses of the basilar arteries to potassium chloride (KCl) and endothelin. Each group consists of six rats. *P <**0.05, **P <**0.01, ***P <**0.001 Two‐way ANOVA.

KCl contracted the basilar arteries in a concentration‐dependent manner with decreased sensitivity and diminished maximal response demonstrated in arteries from aged rats (Fig. 1A). Contractility to KCl was significantly (*P *<**0.05–0.01) greater at the higher doses (120–150 mmol/L) in the 3‐month‐old young rats compared with two older ages with a Log EC_50_ of 80.3 mmol/L, 50.4 mmol/L, and 79 mmol/L, respectively, for 3‐, 8‐, and 24‐month‐old rats. The maximum contractility was similar for 8‐ and 24‐month‐old rats yet considerably lower than the maximum response of the young animals.

There was a decreased sensitivity to the contractile response (*P *<**0.05–0.001) in 24‐month‐old animals, requiring a higher concentration (EC_50_ of 6.39 × 10^−8^ mol/L) to achieve 50% of maximal response as compared to EC_50_ (1.3 × 10^−8^ mol/L) of 3‐month‐old rats and (1.9 × 10^−9^ mol/L) 8‐month‐old rats (Fig. 1B).

### Relaxation responses

**Figure 2.**
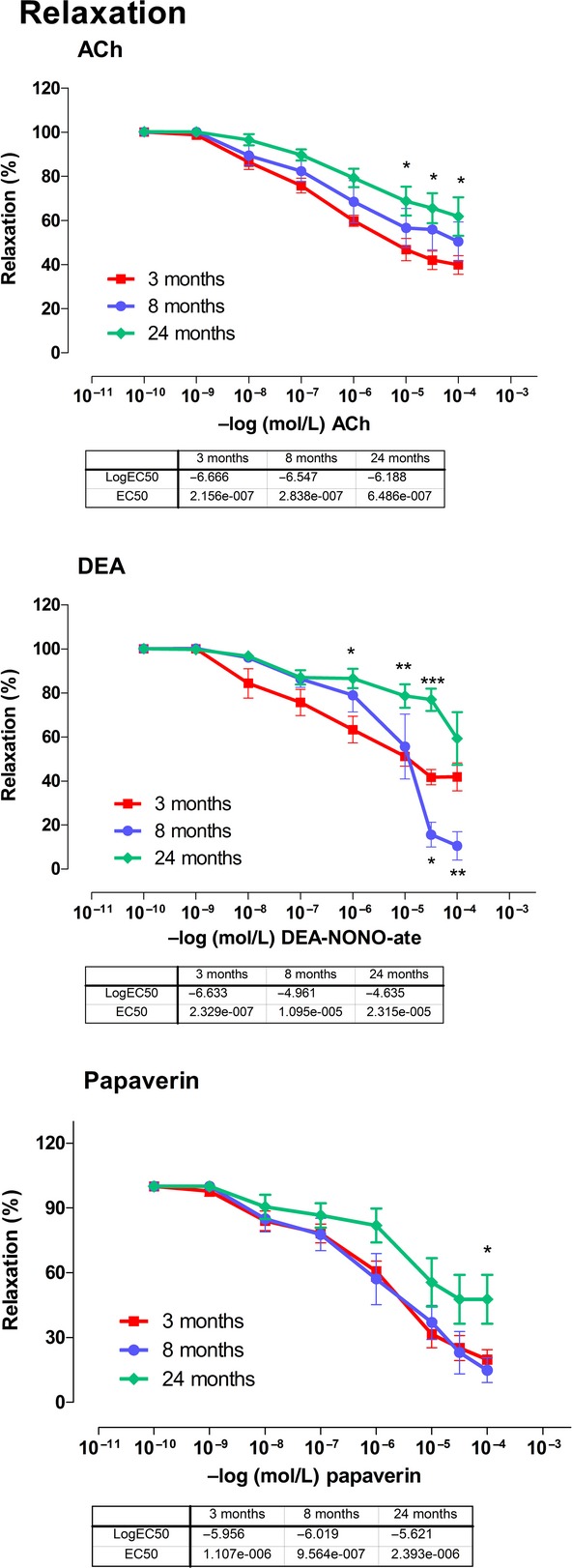
Acetyl choline (ACh), diethyl amine (DEA)‐NONO‐ate and papaverin relaxation of the basilar arteries. Each group consists of six rats. *P <**0.05, **P <**0.01, ***P <**0.001 Two‐way ANOVA.

The relaxation responses to cumulative ACh (10 ^−10^ to 10 ^−4^ mol/L) demonstrated a gradual decrease in sensitivity with age. However, the decrease was only significant (*P *<**0.05) for 24‐month‐old animals. EC_50_ were 2.15 × 10^−7^ mol/L, 2.83 × 10^−7^ mol/L and 6.49 × 10^−7^ mol/L, respectively with increasing age (Fig. 2A).

The NO donor DEA‐NONO‐ate induced relaxation in basilar arteries that was markedly more sensitive (*P *<**0.05–0.001) in the 3‐month‐old young rats when compared with 8‐ and 24‐month‐old animals (EC_50_of 2.33 × 10^−7^ vs. 1.09 × 10^−5^ and 2.31 × 10^−5^ mol/L). On the other hand, maximal vasodilation to DEA‐NONO‐ate was significantly (*P *<**0.05–0.01) augmented at higher concentrations (3 × 10^−5^–10^−4^ mol/L) in 8‐month‐old rats as compared to that in the young and senescent rats (Fig. 2B).

When papaverin was added cumulatively (10^–10^–10 ^−4^mol/L) to basilar rings, only the maximum relaxation response (52.3% vs. 80.3 and 85.1%) was lower in the old animals (Fig. 2C). The EC_50_ values were unchanged across age (1.1 × 10^−6^, 9.6 × 10^−7^, and 2.4 × 10^−6^M for 3‐, 8‐, and 24‐month‐old animals, respectively).

### General characteristics of vessels and histomorphology

The maximal diameter obtained in the calcium‐free buffer and in the presence of 10^−4 ^mol/L SNP was similar across ages at the end of the experiments (Table 1). The spontaneous diameters and wall thickness of 8‐ and 24‐month‐old rats were significantly greater than that of 3‐month‐old rats. However, wall/ lumen ratio was significantly (*P *<**0.05) greater in the 24‐month‐old rats (Table 1).

**Table 1.** General characteristics of the basilar arteries in different age groups

	3 months	8 months	24 months
Maximal diameter (*μ*m)	402.2 ± 17	443.0 ± 14	429.2 ± 15
Spontaneous diameter (*μ*m)	357.0 ± 13	422.5 ± 15*	397.7 ± 16*
Wall thickness (*μ*m)	20.5 ± 0.7	25.9 ± 0.9**	33.3 ± 1.1 ***, ^+++^
Wall/Lumen ratio	0.051 ± 0.002	0.058 ± 0.002	0.078 ± 0.004***,^++^
Wall/adventitia ratio	9.5 ± 1.8	10.1 ± 3.2	12.7 ± 1.4**
Wall/media ratio	1.3 ± 0.2	1.3 ± 0.16	1.2 ± 0.1
Wall/intima ratio	13.6 ± 2.7	13.38 ± 1.53	14.73 ± 2.23
Media/intima ratio	9.9 ± 1.6	10.1 ± 1.4	12.4 ± 0.8**,^++^
Smooth muscle cell count/area	1.6 ± 0.3	1.3 ± 0.3	0.9 ± 0.3**,^+^

*P <**0.05, **P <**0.01, ***P <**0.001 versus 3 months; ^+^*P *<**0.05, ^++^*P *<**0.01, ^+++^*P *<**0.001 versus 8 months.

**Figure 3.**
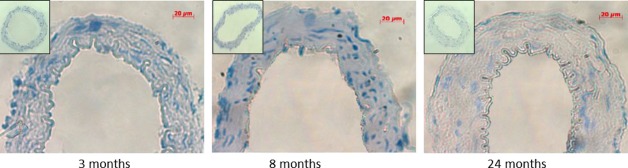
Hematoxylin & Eosin staining of basilar arteries. The number of smooth muscle cells was decreased in the 24‐month‐old group and the wall thickness was increased. (insets: ×200).

When the artery wall was further analyzed by H&E staining (Fig. 3), no change was observed in intima thickness among groups. However, the wall/adventitia and media/intima ratio were significantly (*P *<**0.01) higher in the 24‐month‐old group along with a significant (*P *<**0.01) decline in the number of smooth muscle cell nuclei per area (Table 1).

### Serum antioxidant capacity

**Figure 4.**
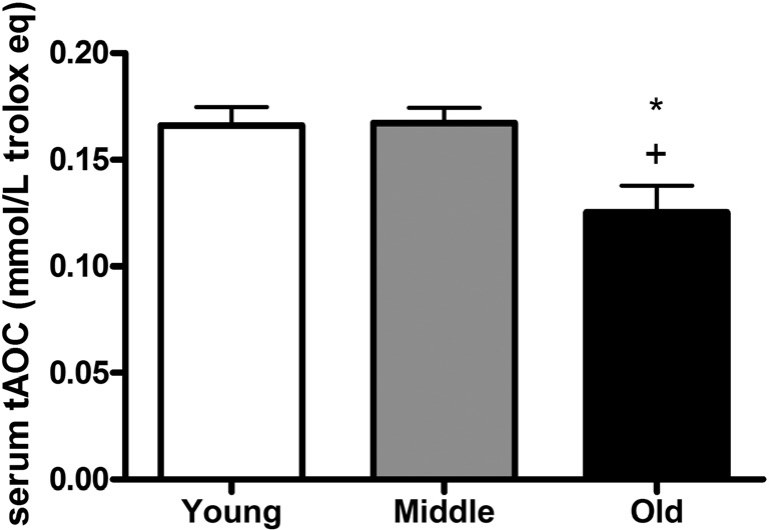
Serum total antioxidant capacity (tAOC) of the 3‐, 8‐, and 24‐month‐old rats (six per group). *P <**0.05 3 months versus 24 months; ^+^*P *<**0.05 8 months versus 24 months.

Serum antioxidant capacity was significantly decreased in 24‐month‐olds when compared with 3‐ and 8‐month‐old rats (Fig. 4).

## Discussion

Vascular smooth muscle cells are the stromal cells of the vascular wall which are involved in the regulation of blood pressure and tissue perfusion in resistance vessels (Lacolley et al. 2012). In this study, our findings demonstrate that the basilar artery wall thickness gradually increased with age. Medial thickness and media/intima ratio were shown to increase in aged human cerebral arteries, indicating smooth muscle hypertrophy and hypertrophic remodeling (Masawa et al. 1994; Gudiene et al. 2011). Our findings demonstrated that wall/ lumen ratio was increased and the maximal diameter tended toward an increase but it was not statistically significant. These data suggest that outward remodeling occurred. In large conduit arteries, remodeling leads to vascular smooth muscle cells that exhibit age‐associated secretory phenotype and lose of contractility. They lose myofilaments and their contractile ability, exhibiting a high rate of proliferation and production of extracellular matrix proteins (Csiszar et al. 2012).

It is known that the increase in the wall thickness and decrease in the smooth muscle cells along with increased collagen network area and decreased elastin function may be responsible for the stiffening of the basilar artery in aging (Hajdu et al. 1990; Fonck et al. 2009) and contribute to the development of atherosclerosis and vertebrobasilar insufficiency. Moreover, vasospasm of the basilar artery is one of the main complications of subarachnoid hemorrhage. Since, the basilar artery is the most important artery in the posterior cerebral circulation, the vasomotor function of this artery is essential for maintaining the regular blood flow to midbrain structures. Moreover, the occlusion of the carotid artery (forebrain ischemia) may also alter the vasoreactivity of the basilar artery. de Andrade et al. (2009) observed that occlusion of the left carotid artery reduced the contractile response of the stenotic carotid artery, whereas the response in the contralateral carotid and basilar artery was increased with endothelin‐1. In our study, we observed smooth muscle hypertrophy and decreased contractility to ET‐1 and KCl, which is potentially linked to vascular smooth muscle hypertrophy related to changes in the contractile ability.

There are a number of studies which report that aging impairs contractility and/or relaxation in coronary arteries and other peripheral vessels (Ishida et al. 2003). However, there are limited numbers of studies with cerebral vessels. The endothelial dysfunction in the cerebral vessels and decreased blood flow may be a precipitating factor for neurological disorders that vary from vertigo, headaches, sleep disturbances, pupillary and oculomotor abnormalities, dysarthria, and dysphagia to quadriparesis. Endothelial dysfunction is characterized by decreased production and/or bioavailability of nitric oxide (NO). According to the free radical hypothesis of vascular aging, excessive generation of reactive oxygen species leads to increased formation of peroxynitrite (formed by the binding of superoxide with NO) which activates prostaglandin metabolism and age‐related vascular remodeling (Bachschmid et al. 2013). These changes further lay the groundwork for cardiovascular diseases (Muller‐Delp 2011; Muller‐Delp et al. 2012). In this study, we found that serum antioxidant capacity was significantly decreased in the oldest group. Plasma antioxidant capacity is one factor that contributes to the plasma oxidative stress. Plasma oxidative stress index correlates with the tissue oxidative stress index during chronic diseases in humans (Rabus et al. 2008). Therefore, plasma antioxidant capacity can potentially affect the oxidative stress in the peripheral tissues. Moreover, it was previously reported that carotid atherosclerosis was associated with the reduced serum antioxidant capacity (Skalska and Grodzicki 2010). Accordingly in this study, decreased plasma antioxidant capacity may be an indicator of increased oxidative stress in the basilar artery, the latter which leads to structural and functional impairment. In particular, reduced endothelium‐dependent vasodilation to ACh may be the result of increased oxidative stress.

ACh‐induced endothelium‐dependent relaxation is largely attributed to NO, whereas endothelium‐independent relaxation is likely to be mediated by ion channels. Ion channels, especially potassium channels, expressed in both endothelial and vascular smooth muscle cells are critical for the maintenance of vascular tone (Nelson and Quayle 1995). More specifically, vascular smooth muscle cells express at least four different types of potassium (K^+^) channels, one or two types of voltage‐gated calcium (Ca^2+^) channels, at least two types of chloride (Cl^−^) channels, store‐operated Ca^2+^, channels, and stretch‐activated cation channels in their plasma membranes, all of which may be involved in the regulation of vascular tone. Experimental evidence indicates that at least one subtype of K^+^ channel, the BK channel (the large‐conductance Ca^2+^‐activated K^+^ channel) has its expression/function decreased with aging (Carvalho‐de‐Souza et al. 2013). Our data indicate that in addition to the decreased KCl‐induced contraction, endothelium‐dependent and ‐independent relaxation of the smooth muscle are impaired with age in the basilar artery, and this decline in smooth muscle responsiveness is consistent with the report of reduced K^+^ channel function/expression with age.

Aging not only impairs the relaxation responses but also may decrease vascular smooth muscle contractility. Previous studies reported that vasoreactivity of small arteries to angiotensin II and ET are altered in aged rats, although their blood pressure and heart rates remain unchanged (Moreau et al. 1998). Maximal contraction caused by KCl, norepinephrine, and 5‐hydroxytryptamine (5‐HT) decreased with age in basilar arteries from human cadavers (Hatake et al. 1992). Also, in another study ET, phenylephrine‐, and KCl‐induced contractions were reduced in basilar and carotid arteries of guinea pigs (de Andrade et al. 2009). On the other hand, some other researchers have reported that there are no age‐related differences in KCl or 5‐HT contractions of rat cerebral arteries, whereas significant decreases in endothelium‐dependent relaxation to bradykinin or norepinephrine were observed (Arribas et al. 1997).

Recent studies focused on the role of angiotensin II in cerebral vessels. Toth et al. (2013) found autoregulatory dysfunction of the cerebral vasculature with angiotensin II‐induced hypertension in aged mice. In another study, losartan (angiotensin II type 1 receptor antagonist) was shown to have a dose‐dependent antagonistic effect to ET‐induced constriction, possibly via ET_B_ receptors (Konczalla et al. 2013). However, the MAPK pathway has been demonstrated to be involved in the ET_A_‐mediated contractile responses in the basilar arteries of rabbits (Zubkov et al. 2000). Such crosstalk was also shown between alpha‐1 adrenoreceptors and angiotensin receptors; where activation of AT_1_ receptors enhanced the positive inotropic response induced by the activation of alpha‐1 adrenergic receptors in the atria of aged rats (Li and Shi 2009). However, these effects may vary among species. In our experiments, we used F344X BN rats and were unable to obtain a dose–response curve with Angiotensin II in rat basilar arteries (data not shown). The results demonstrated, however, a decrease in the contractile response to ET and KCl in the basilar arteries of 24‐month‐old rats when compared to arteries from 3‐month‐old rats.

Modrick et al. (2009) reported that cerebral vascular responses to ACh were reduced by ∼50% in old wild‐type mice but were normal in old AT_1_‐deficient mice, whereas the responses to papaverin response were unaltered. Consistent with this study, we found that both endothelium‐dependent relaxation responses to ACh and relaxation responses to the NO donor DEA‐NONO‐ate were decreased. ACh‐induced relaxation is mediated by NO that is released from the endothelium, whereas DEA‐NONOate provides the NO exogenously even though endothelium function is impaired. Interestingly, the maximal relaxation obtained with DEA was significantly higher in 8‐month‐old rats, possibly indicating an age‐related optimization. However, the EC_50_ values were similar for 8‐ and 24‐month‐old rats and significantly higher than that for the 3‐month‐old rats. The maximal response to papaverin was significantly lower in the old rats, although the EC_50_ values were not different among groups. Papaverin is a direct relaxant of the smooth muscle and its mechanism of action is thought to be mediated via cAMP. The age‐related decrease in papaverin response was previously reported in coronary arteries (Hatake et al. 1992) and tracheal smooth muscle cells (Preuss and Goldie 1999; Tian et al. 2011). Considered together, our data suggest that both endothelial and vascular smooth muscle mechanisms of vasodilation are impaired with age. Such changes in the vasodilator properties of the basilar artery could contribute to the diminished perfusion and higher vascular resistance in the posterior cerebral circulation of old rats at rest (Ohata et al. 1981; Salter et al. 1998) and during periods of stress, such as under environmental conditions of hypoxia and hypercapnea (Haining et al. 1970). Further experiments will be needed to define the intracellular mechanisms that underlie the age‐related reduction of vasodilatory responses in the basilar artery.

To our knowledge, this is the first report showing both decreased serum antioxidant capacity and impaired age‐related changes in the basilar artery reactivity and structure in F344XBN rats, an accepted model of aging. These decreased relaxation of the smooth muscle cells could be either be cAMP or cGMP mediated. Herein, we demonstrate that endothelium‐dependent and ‐independent basilar artery relaxation were impaired with age, suggesting a deficient NO/cGMP‐mediated mechanism rather than cAMP. This conclusion is reinforced by the nearly unchanged papaverin relaxation that is mainly mediated by cAMP. Moreover the decreased responses were accompanied by structural remodeling and decreased antioxidant capacity of the serum. Thus, this study provides a mechanistic insight for the aging‐induced vascular dysfunction of the basilar arteries.

## Conflict of Interest

None declared.
